# Achievements of the British Women's Hospital Committee

**Published:** 1921-02-19

**Authors:** 


### Achievements of the British Women's Hospital Committee.

The concise summary of the work accomplished by the British Women's Hospital Committee from July 1915 to December 31, 1919, which has been recently issued,* is a chapter of war history at once romantic and exhilarating. The Committee began by shouldering the task of raising £50,000 for the purpose of building a great institution for totally disabled sailors and soldiers on the site of the Star and Garter Hotel, at Richmond. The Chairman of the Actresses' Franchise League, now Dame May Whitty, was appointed Chairman and Viscountess Cowdray Hon. Treasurer of this fund, which in the end reached the magnificent total of £223,947.

The next movement to enlist the powerful support of the British Women's Hospital Committee was the Scottish Women's Hospitals. The sum of £12,971 was handed over to this organisation, while £500 was given to the Lord Roberts Memorial Fund and £500 to the Hampstead General Hospital.

In October 1917 the British Women's Hospital Committee launched a third appeal, this time in

* To be obtained from the Secretary of the Nation's Fund for Nurses, 32 North Audley Street, London, W. 1. Price 1s., or 1s. 2d. post free.

the interests of nurses. The aim was twofold—first, the endowment of the College of Nursing, to raise the standard of nursing education and to be a permanent bulwark of defence for British nurses; secondly, the rescue of individual nurses from the distress incurred by sickness, disablement, and old age. The marked feature of the appeals issued to the public for these purposes was the whole-hearted support extended to them by the leaders of the nursing profession, no less than by nurses themselves. Our readers have from time to time followed with deep interest the successful issue of many efforts made on their behalf by the British Women's Hospital Committee, but particulars of the sum total realised are now for the first time, owing to unavoidable delay in passing special grants through the accounts, presented to the public.

The statement of receipts and payments relating to the Nation's Fund for Nurses covers the period from June 6, 1917, to December 31, 1919. At this latter date the British Women's Hospital Committee handed over the net results of their efforts on behalf of nurses to the Council and Executive of the Nation's Fund for Nurses, whose report of last year's development will be published in due course. What, then, did the British Women's Hospital Committee achieve for the nurses of Great Britain?

For the College of Nursing the sum of £35,650 has been secured for endowment, while £556 has been invested for scholarships. The London Centre of the College has received a grant of £3,000. For the Tribute Fund, now administered by a sub-committee of the Nation's Fund for Nurses, the sum of £69,967 (including the Red Cross grant of £50,000) has been invested for grants and pensions to necessitous nurses. The sum of £10,037 collected in Ireland has been returned to meet the needs of Irish nurses by local administration. Other sums bring the grand total to £148,915, of which only £8,087—about 5 per cent.—has been absorbed in expenses. When it is remembered that the receipts include the sum of £33,505 from entertainments, at a cost of less than 3 per cent., some slight measure of the self-sacrifice and good business methods of the promoters may be realised.

It is sufficient to say of this great movement, initiated by the British Women's Hospital Committee with enthusiasm, and carried out with singular patience, foresight, and energy, that it has opened a new era for the nurses of Great Britain. What chance, it may be asked, had nurses, overburdened with national duties during the years covered by these activities on their behalf, of raising unaided the funds necessary to start the College of Nursing? To glance over the early reports of the College is to become aware of the great cost involved in launching an enterprise of this magnitude. When, side by side with this, it became necessary to provide for nurses broken down in health and to ensure the permanence of undertakings which involved the future well-being of the profession, it can be seen how hopeless a task lay before the nursing profession until the British Women's Hospital Committee came generously to the rescue and lifted the burden of finance off their sisters' shoulders.

For many years to come there will be a close bond of sympathy between the members of two widely different professions, who were brought together on common ground, with a common impulse of patriotism, in the darkest days of the Great War.

